# Glioblastoma may evade immune surveillance through primary cilia-dependent signaling in an IL-6 dependent manner

**DOI:** 10.3389/fonc.2023.1279923

**Published:** 2023-12-18

**Authors:** Maxwell T. Laws, Erin N. Walker, Francesca M. Cozzi, Leonel Ampie, Mi-Yeon Jung, Eric C. Burton, Desmond A. Brown

**Affiliations:** ^1^ Neurosurgical Oncology Unit, Surgical Neurology Branch, National Institutes of Neurological Disorders and Stroke, National Institutes of Health, Bethesda, MD, United States; ^2^ University of South Carolina School of Medicine Greenville, Greenville, SC, United States; ^3^ Cambridge Brain Tumour Imaging Lab, Division of Neurosurgery, Department of Clinical Neurosciences, University of Cambridge, Addenbroke’s Hospital, Cambridge, United Kingdom; ^4^ Neuro-Oncology Branch, Center for Cancer Research, National Cancer Institute, National Institutes of Health, Bethesda, MD, United States

**Keywords:** glioblastoma, extracellular vesicles, primary cilia, glioblastoma-mediated immunosuppression, IL-6, CCRK

## Abstract

Glioblastoma is the most common, malignant primary brain tumor in adults and remains universally fatal. While immunotherapy has vastly improved the treatment of several solid cancers, efficacy in glioblastoma is limited. These challenges are due in part to the propensity of glioblastoma to recruit tumor-suppressive immune cells, which act in conjunction with tumor cells to create a pro-tumor immune microenvironment through secretion of several soluble factors. Glioblastoma-derived EVs induce myeloid-derived suppressor cells (MDSCs) and non-classical monocytes (NCMs) from myeloid precursors leading to systemic and local immunosuppression. This process is mediated by IL-6 which contributes to the recruitment of tumor-associated macrophages of the M2 immunosuppressive subtype, which in turn, upregulates anti-inflammatory cytokines including IL-10 and TGF-β. Primary cilia are highly conserved organelles involved in signal transduction and play critical roles in glioblastoma proliferation, invasion, angiogenesis, and chemoradiation resistance. In this perspectives article, we provide preliminary evidence that primary cilia regulate intracellular release of IL-6. This ties primary cilia mechanistically to tumor-mediated immunosuppression in glioblastomas and potentially, in additional neoplasms which have a shared mechanism for cancer-mediated immunosuppression. We propose potentially testable hypotheses of the cellular mechanisms behind this finding.

## Introduction

Glioblastoma is the most common malignant brain tumor with mean survival of 15 months, and average 5-year survival of 6.9% (1). Despite significant effort to develop novel therapies, there has been little improvement in outcomes. The pathogenesis of glioblastoma involves myriad cellular adaptations which promote proliferation, invasion, angiogenesis, DNA repair, and immune suppression. Immunotherapies have garnered significant interest among the scientific community and have revolutionized treatment of several solid cancers. Glioblastoma is notorious for propagating an immunosuppressive tumor microenvironment (TME) through suppressing infiltrating immune cells via numerous pathways which work both locally and systemically. In the local tumor microenvironment, production of tryptophan metabolites, secretion of cytokines including IL-6 and IL-10, and an increase in membrane expression of checkpoint proteins like PD-L1 result in local immunosuppression. Contemporary evidence also implicate glioblastoma-derived extracellular vesicles (EVs) in upregulation of myeloid-derived suppressor cells (MDSCs) which contribute to systemic immunosuppression (2–5). While it is known that glioblastoma generates an immunosuppressive TME, the specific alterations in gene expression and cellular signaling which trigger this immunosuppressive phenotype remain enigmatic.

Primary cilia are non-motile, microtubule-based organelles which act in key signaling pathways, e.g. EGFR (6), Shh (7), WNT (8), TGF (9) and Notch (10). The primary cilium is anchored to the plasma membrane by the basal body. The basal body is important because it acts as a template for cilia construction and repurposes itself in cell division as the mother centriole (11). The implication of this juxtaposition with the centrosome, is that the primary cilia must be disassembled before the cell can transition from the G_0_/G_1_ phase to the cycling S/G_2_/M phase of mitosis (12). This “ciliary checkpoint” acts as a brake, confining the cell to the G_0_/G_1_ phase. Perhaps unsurprisingly, many systemic and CNS malignancies including glioblastoma, melanoma, pancreatic, liver, and prostate cancers demonstrate reduction in primary cilia frequency, though in each of these tumors, a ciliated cell population remains (13, 14). In this perspectives article, we present evidence for a potential role of primary cilia as master regulators of glioblastoma-mediated immunosuppression through the regulation of IL-6. This is a novel idea which may ultimately yield deeper understanding of the pathogenesis of glioblastoma and other cancers which all rely on this shared mechanism and may also allow for development of potentially novel and urgently needed therapeutic strategies.

## Methods

### Source of human glioblastoma cells and cell culture

Human glioblastoma cells (dBT114 and dBT116) were acquired from the Brain Tumor PDX National Resource Database by Sarkarias et al. from the Mayo Clinic (Mayo Clinic IRB312-003458). Cells were cultured in DMEM/F12 (Thermo Fisher Scientific, Waltham, MA) with 10% fetal bovine serum (FBS) and 1% Pen/Strep and incubated in 5% CO_2_ at 37 degrees Celsius.

### Protein knockdown by small interfering RNA

siRNA sequences targeting KIF3A (sc-270301), CCRK (sc-92544), IFT88 (sc-75329), or a nontargeting siRNA control (sc-37007) were acquired (Santa Cruz Inc, Dallas, TX). Each siRNA product consisted of pools of 3–5 target-specific 19–25 nucleotide siRNAs designed to knock down expression of the gene of interest. Glioblastoma cells (2.5 × 10^5^) were incubated in DMEM with 10% FBS and siRNAs were transfected using Lipofectamine RNAiMAX (Thermo Fisher Scientific) per the manufacturer’s instructions. Cells were then recovered in complete medium for 24 hours, and the efficacy of gene targeting at the mRNA and protein level was assessed by qRT-PCR and western blotting, respectively.

### Immunoblotting and densitometric analysis

Whole-cell lysates (WCL) were prepared with RIPA buffer (50 mM Tris [pH 7.4], 1% Triton X-100, 0.25% sodium deoxycholate, 150 mM NaCl, 1 mM EDTA [pH 8], and 10 mM NaF) containing complete Protease Inhibitor (Roche, Basel, Switzerland). Protein concentration was determined with a BCA assay (Thermo Fisher Scientific) per the manufacturer’s instructions. Proteins were then separated by electrophoresis on NuPAGE 4-20% gradient precast polyacrylamide gels (Life Technologies, Carlsbad, CA). Following membrane transfer, the proteins were probed using the following antibodies: HSP90 (4874S; Cell Signaling, Danvers, MA), KIF3A (PA5-121019), IFT88 (PA5-18467), CCRK (PA5-52464), and IL-6 (M620) all sourced from Thermo Fisher Scientific. Secondary antibody was horseradish peroxidase-conjugated goat anti-rabbit (Jackson ImmunoResearch, West Grove, PA). Detection was enhanced by chemiluminescence. Immunoreactive band density was then quantified using ImageJ software (NIH, Bethesda, MD).

### RNA extraction and qRT-PCR

Total RNA was isolated from glioblastoma cells (3 x 10^5^) using the RNeasy Plus Mini Kit (Qiagen, Valencia, CA). Isolated RNA (500 ng) was then utilized to perform a reverse-transcription reaction (30 μl) with random hexamers and SuperScript III RT (Thermo Fisher Scientific). The resulting cDNA (5 μl) was used for real-time PCR using the TaqMan gene-expression assay for KIF3A (Hs00199901_m1), CCRK (Hs01114921_m1), IFT88 (Hs00544051_m1), and actin (Hs00188792_m1), according to the manufacturer’s instructions. 2−ΔΔCt was used to determine the relative expression levels of the target genes. All experiments were performed in triplicate.

### Enzyme-linked immunosorbent assay

The cellular levels of IL-6 were measured after knockdown of cilia proteins using ELISA. Specifically, the Millipore Human IL-6 ELISA kit (Millipore, Billerica, MA, Cat#RAB0306) was performed according to the manufacturer’s instructions and analyzed at 450 nm using a plate reader (BioTek, Winooski, VT). Each sample was performed in triplicate from which the means and standard deviations were calculated.

### Immunocytochemistry

Transfection of dBT116 was conducted by transferring 10 µL of shRNA transduction particles (Clone ID TRCN0000199977, SHCLNV, Sigma-Aldrich, St. Louis, MO) in polybrene (10 µg/mL) to a 6 well plate containing 1 x 10^6^ bone marrow mononuclear cells in 3 mL of complete dBT116 cell medium. After 24 hours, the medium was changed to virus-free complete DMEM with 10% FBS medium, and puromycin selection was initiated (2 µg/mL, Sigma-Aldrich). Immunocytochemistry experiments were conducted on days 5-7 after antibiotic selection.

Two-well chamber slides were coated with collagen, type I solution (Sigma Aldrich, Burlington, MA) diluted in 70% ethanol per manufacturer protocol. Cultured cells were seeded and incubated overnight at 37 degrees Celsius. Cells were washed in filtered 1X PBS three times and fixed with 4% formaldehyde for 10 minutes at room temperature. Cells were washed three times in filtered 1X PBS, permeabilized in filtered 0.5% Triton X-100 in PBS for 15 minutes, washed four times and incubated with filtered blocking solution (PBS containing 0.15% Glycine and 0.5% BSA) for 60 minutes at room temperature. Cells were then incubated with primary antibodies in 1X PBS for 1 hour, washed three times with 1X PBS, and incubated in the dark with secondary antibodies conjugated to Alexa Fluor 488 and 549 for Arl13B and gamma tubulin, respectively, in 1X PBS for 1 hour at room temperature (1:300, Invitrogen, Waltham, MA). Primary antibodies used were mouse monoclonal Arl13B (1:300, Invitrogen), rabbit monoclonal gamma tubulin (1:300, Invitrogen). Antifade mounting medium with DAPI was used to coverslip the slides (Thermo Fisher, P36935). Images were collected using a Leica DMi8 widefield fluorescence microscope with a 20X objective for DAPI, Alexa 488, and Alexa 549 fluorophores (Leica Biosystems, Buffalo Grove, IL).

### Statistical analysis

All data represent at least three individual experiments. For the direct comparison of three or more conditions a one-way analysis of variance was performed, with multiple comparisons analyzed via Newmans-Keuls multiple comparisons test. When directly comparing two conditions a two-tailed student-t test was performed. All comparisons were considered significant with p-values less than 0.05.

## Results

### Primary cilia loss reduces IL-6 expression in human glioblastoma cells

**Figure 1:**
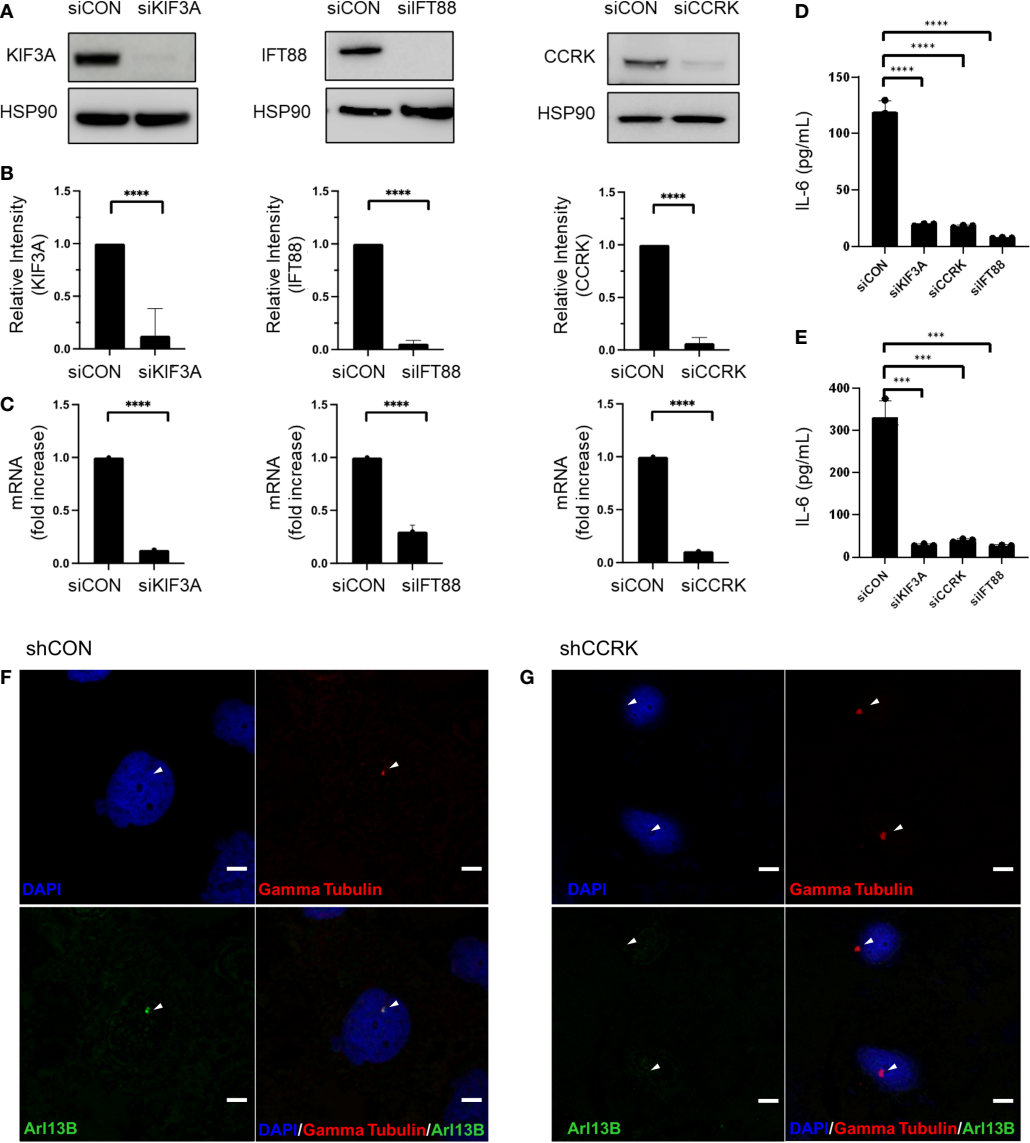
IL-6 expression is dependent on primary cilia. Ciliogenesis-required proteins KIF3A, IFT88, and CCRK were depleted via transfection using *siRNA* or a scrambled control (*siCON*). **(A)** dBT114 cell protein levels were assessed with immunoblotting assay with HSP90 serving as loading control. **(B)** Densitometric analysis of the bands are shown. **(C)** The effect of knockdown on *IL-6* mRNA transcription in dBT114 cells was assessed by qRT-PCR. Knockdown of genes required for ciliogenesis resulted in depletion of IL-6 as assessed by ELISA for cell lines **(D)** dBT116 and **(E)** dBT114. All experiments were performed in triplicate. Means were compared using the two-sided student’s t-test. *** denotes a P-value <0.005. CCRK was suppressed using shRNA or a scrabbled control (shCON) and immunocytochemistry performed on dBT116 glioblastoma cells for the basal body with gamma tubulin and then primary cilia axoneme with Arl13B. Nuclei were stained with a DAPI counterstain. Representative images were obtained at 20X with DAPI, Alexa 488, and Alexa 549 fluorophores. Scale bar 20 µm. **(F)** Control dBT116 cells (shCON) demonstrating the presence of a basal body as well as an adjoining Arl13B-positive axoneme indicating the presence of a primary cilia. **(G)** CCRK suppressed dBT116 cells (shCCRK) have basal bodies revealed through gamma tubulin staining, but lack primary cilia, as evidenced by the absence of a Arl13B-positive axoneme. Arrows denote structures of interest. **** denotes a P-value <0.001.

The function of primary cilia in cancer has been gaining interest over the past two decades, and is known to influence pathogenesis of some CNS neoplasms including medulloblastoma, choroid plexus papilloma, and ependymoma (15). *In vitro* and *in vivo* models of ciliary ablation including knockdown or knockout of *KIF3A* and *IFT88* have been invaluable tools for dissecting and understanding the myriad functions of the primary cilium. *KIF3A* is a microtubule plus end-directed kinesin motor that is required for ciliogenesis (16). Intraflagellar transport protein 88 (IFT88) is necessary for primary cilia assembly via the transport of essential components up the ciliary axoneme (17). Previous immunocytochemistry studies have shown loss of primary cilia following KIF3A or IFT88 knockdown (18, 19). Cell cycle-related kinase (CCRK) now known as CDK20 has been specifically implicated in tumor-mediated immunosuppression by induction of MDSC in response to IL-6 upregulation (20). However, we previously showed that CCRK is required for proper cilia morphogenesis in a knockout mouse model (21). As CCRK is highly overexpressed in glioblastoma and considered an oncogene, we questioned whether the elevated IL-6 expression and the resulting immunosuppressive phenotype was due to CCRK’s role in cilia morphology versus other unrelated activity. To that end, we performed siRNA-mediated knockdown of *CCRK* as well as *KIF3A* and *IFT88*. Our hypothesis was that if IL-6 expression was dependent on primary cilia, then all three knockdowns would result in reduction of IL-6 expression. As demonstrated in **Figure 1**, siRNA targeted against *KIF3A*, *IFT88*, and *CCRK* resulted in robust knockdown of each protein (**Figures 1A, B**) as well as each corresponding mRNA (**Figure 1C**). Fluorescence immunocytochemistry confirmed primary cilia loss with CCRK suppression compared to control (**Figures 1F, G**). Loss of these essential ciliary genes each resulted in a similar loss of IL-6 protein levels through ELISA (**Figures 1D, E**).

## Discussion

Glioblastoma remains a universally incurable and fatal disease. One important characteristic of glioblastoma is profound local and systemic immunosuppression. The latter is the result of a multitude of means by which glioblastoma hijacks the immune system including the kynurenine-tryptophan (IDO-TDO1) pathway, expression of pro-mitogenic, immunosuppressive EVs, the release of anti-inflammatory cytokines, and manipulation of checkpoint proteins (e.g., PD-1, CTLA-4). There is considerable interest in studying the biological roles of primary cilia in glioblastoma as a path for drug development. Prior studies established that cilia are present in glioblastoma cells, with one study finding 8-25% of glioblastoma cells bearing primary cilia at any point in time (22). The same group later found that 60-90% of single clones from patient-derived glioblastoma cell lines were able to generate ciliated offspring (23). While cilia-dependent signaling is present in glioblastoma, it is unclear how cilia-dependent signaling cascades act in a tumor-promoting or suppressing manner. For instance, disruption of cilia formation in glioblastoma cell lines through knockdown of essential ciliogenesis genes, such as KIF3A or IFT88, had variable effects on tumor growth *in vitro* and *in vivo* (23). There is evidence, however, that treatments aimed to reduce ciliogenesis could enhance conventional glioblastoma therapies. For instance, PCM1-mediated depletion of cilia in patient-derived glioblastoma cell lines led to decreased proliferation and increased sensitivity to temozolomide (TMZ) treatment (24). There is a preponderance of evidence that now links canonical pathways of glioblastoma immune evasion with primary cilia signaling. The objective of this perspectives article is to review evidence supporting potential mechanisms by which cilia-dependent signal transduction contributes to glioblastoma-mediated immunosuppression.

### CCRK, IL-6 and the cilia connection

Cell cycle-related kinase (CCRK) plays an evolutionarily conserved role in the assembly of cilia and is highly overexpressed in gliomas where it is thought to play an oncogenic role (21, 25). CCRK knockout mice display neural tube and skeletal defects identical to those seen in SHH deficient mice; embryonic fibroblasts derived from these mice showed dysmorphic, non-functional cilia (21). *In vitro*, CCRK overexpression reduces cilia frequency and promotes proliferation in the U-251 glioblastoma cell line (26). Conversely, CCRK silencing led to the inhibition of cell growth in high CCRK-expressing U-373 and U-87 cell lines (27). Interestingly, CCRK activity has been linked to cytokine expression in other tumor models. For instance, in the Hepa1-6 hepatocellular carcinoma model, CCRK is necessary for IL-6 expression which led to the expansion of MDSCs in peripheral blood (20). Whether this relationship between IL-6 and CCRK was related to the role of the latter in cilia structure and function has never been explored. We now show a similar statistically significant reduction in IL-6 intracellular concentrations following depletion of proteins required for ciliogenesis (**Figure 1**). The implication is that it is the primary cilia specifically, and not CCRK per se, that is important in driving IL-6 expression and that the relationship noted between CCRK, IL-6, and MDSC expansion is a direct result of the role of CCRK on primary ciliogenesis. We found a similar result with stable lentiviral transduction of shRNAs against essential ciliogenesis proteins and subsequently performed transcriptomic sequencing (data not shown). A potential mechanism in which cilia signaling may regulate IL-6 expression may be through the GLI1-SHH pathway–the best described cilia-dependent signaling cascade. The binding of SHH to the patched-1 receptor leads to the translocation and accumulation of Smoothened at the ciliary tip and activation of the GLI family of transcription factors, including GLI-1 (28). In a murine model of pancreatic cancer, GLI-1 binds to the IL-6 promoter and increase its expression, leading to a more aggressive phenotype (29). In the absence of activated GLI-1, mice developed only low-grade lesions and at a low frequency. Glioblastoma and pancreatic adenocarcinoma are reliant on EVs and IL-6 for immune modulation and cellular proliferation. Thus, the implication of primary cilia may have far-reaching implications across multiple cancers.

IL-6 has emerged as a potential therapeutic target in treatment of glioblastoma. Rolhion et al. found that glioblastomas displayed significantly higher IL-6 expression compared to other glioma types (3). IL-6 then orchestrates recruitment of tumor-associated macrophages of the M2 suppressive phenotype which produce anti-inflammatory cytokines like IL-10 and TGF-β, which in turn inhibit tumor-associated T-cell invasion and activation (30). Glioblastoma secretion of IL-6 increased PD-L1 expression on peripheral myeloid cells, promoting T cell anergy (31). Levels of IL-6 found in serum and cerebrospinal fluid corresponded to glioma grade, with significant reduction in levels following resection (32). Yang et al. found that knockout of IL-6 reduced the intra-tumoral population of myeloid cells and macrophages and enhanced the population of CD8+ T cells (30). Concomitantly, anti-IL-6 therapy improved overall survival by 30% in a GL261 murine glioblastoma model (30). Analysis of the TCGA dataset revealed that IL-6 and IL-6R mRNA levels were significantly higher in mesenchymal subtype and IDH-wildtype glioblastoma (33). As mentioned previously, the mesenchymal subtype has the highest infiltration of immune cells. The influx and subsequent reprogramming of resident immune cells is due in part to EVs and IL-6, both of which are likely dependent on cilia signaling.

### Review of glioblastoma-mediated immunosuppression: EVs, MDSCs, and tumor-associated myeloid cells

Extracellular vesicles (EVs) are a heterogeneous group of lipid membrane-enclosed vesicles released ubiquitously from cells and contain proteins, nucleic acids, and other biological mediators (34). They allow for intercellular communication in both physiologic and pathophysiologic states. Several cancers including breast, pancreas, prostate, and brain produce high levels of EVs which operationalize the local cellular milieu (35–37). Glioblastoma-derived EVs were first described by Skog et al. in 2008 and actively promote glioblastoma cell proliferation and angiogenesis (38). There is now an abundance of contemporary evidence supporting a role for glioblastoma EVs in regulating multiple pathways that ultimately contribute to several key glioblastoma characteristics including tumor-mediated immunosuppression. Hoang-Minh et al. demonstrated that glioblastoma primary cilia produce vesicles that may have overlap with EVs. During G_0_ phase, glioblastoma primary cilia had vesicles that appeared to bud from the tip and floated away out of the field of view (15). Furthermore, they found that these vesicles had mitogenic capacity, as their presence promoted tumor cell proliferation. It is possible that these same cilia-derived vesicles also contribute to the local and systemic immunosuppression which are hallmarks of glioblastomas and other cancers.

Local immunosuppression in the glioblastoma microenvironment is dependent on tumor-associated myeloid cells (microglia, macrophages, and monocytes) which constitute up to 30-50% of cells within glioblastoma tissues (39–41). These immune cells migrate via chemotaxis into the glioblastoma tumor microenvironment. Once within the tumor stroma, cells in the tumor microenvironment (including immune cells) are exposed to high EV levels and (and presumably EV content) as well as other soluble factors. Glioblastoma EVs in the tumor microenvironment then stimulate local astrocytes to produce cytokines including CSF2 and 3, IL-4, -6, -10, and -13, which together promote a T-helper type 2 immunosuppressive phenotype (42). The result is immune cell reprogramming into immunosuppressive regulatory cells. Furthermore, glioblastoma EVs enhance the phagocytic capacity of tumor-associated macrophages and enhance the expression of membrane type 1-matrix metalloproteinase in microglia (43). Tumor-derived EVs promote extracellular matrix remodeling (ECM), thus facilitating tumor migration and invasion (44, 45). There is evidence of heterogeneity in EV expression and effects among glioblastoma subtypes. Mesenchymal glioblastoma cells secrete EVs at higher levels compared to those of the classical and pro-neural subclass as identified by mass spectroscopy (43). Low glioma EV concentrations are associated with immune activation and increased migration capacity of peripheral blood mononuclear cells (PBMCs), while high EV concentrations impair PBMC migration (46). The mesenchymal glioblastoma subtype has been associated with the highest infiltration of tumor-associated lymphocytes (47).

Glioblastoma-derived EVs are also implicated in systemic immunosuppression in glioblastoma. These EVs induce monocytes into myeloid-derived suppressor cells (MDSCs) and nonclassical monocytes (NCMs). Elevated levels of MDSCs are described in a number of cancers including melanoma, renal, gastric, bladder, pancreatic, and gliomas (48). The induction of MDSCs is particularly robust in glioblastomas with circulating MDSCs in glioblastoma patients estimated at up to 12 times greater than that seen in controls (49, 50). Predictably, there are resident NCMs and MDSCs identifiable in freshly resected glioblastoma tissue (5). Jung et al. showed that induction of MDSCs and NCMs was dependent on both PD-L1 and IDO1 expression within the EVs in a mechanism dependent on interferon-γ (51). Interestingly, there was no identifiable direct effect of glioblastoma EVs on T cells. Instead, there was evidence for production of IL-10 by MDSCs and NCMs with resulting T cell inhibition. Glioblastoma patients have higher proportions of tumor-infiltrating regulatory Tregs – an effect of high circulating MDSCs (52, 53). Compared to PBMCs, the ratio of exhausted CD4+ and CD8+ T cells are significantly higher in tumor regions (54). Glioblastoma-infiltrating NK cells show significantly lower cytolytic ability, owing to lower levels of interferon-γ (54). Treg-depletion in a murine glioma model revealed prolonged survival compared to control mice (55). EVs regulate additional key tumor characteristics including remodeling innate and adaptive immune cell behaviors, promoting therapy resistance, glioma stemness, and tissue invasion.

**Figure 2:**
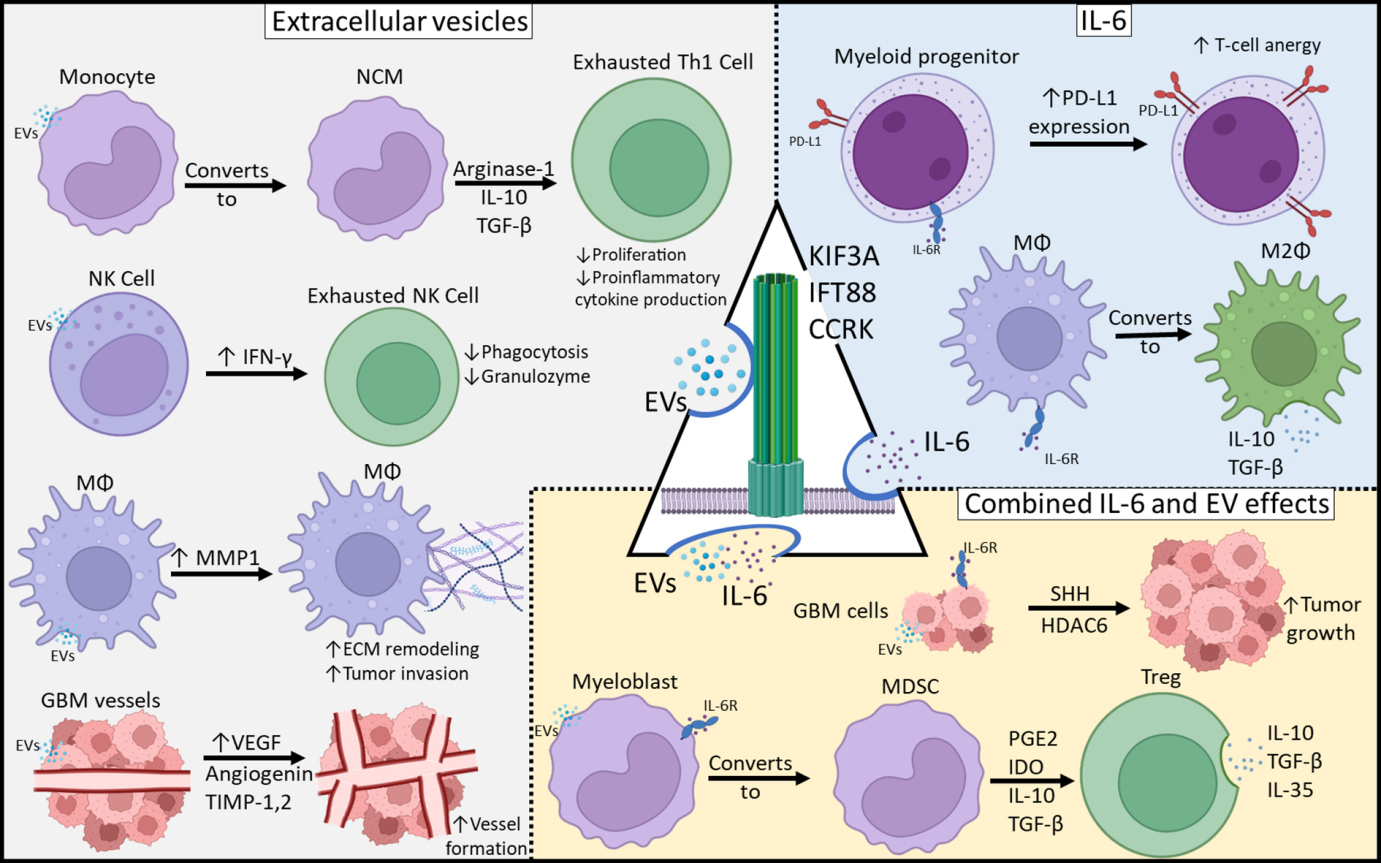
Mechanisms of Glioblastoma-Mediated Immunosuppression. Immunosuppressive effects can be categorized as those resulting from EVs, IL-6 or both. Primary cilia may be involved in both EV release as well as IL-6 expression and those may play a central role in tumor-mediated immunosuppression.

We present a theoretical framework in which primary cilia participate in glioblastoma immune programming (**Figure 2**) and to present preliminary data supporting this hypothesis. We have shown that through disruption of primary cilia via knockdown of CCRK, KIF3A, or IFT88, there is decreased IL-6 protein expression. It is evident that IL-6 is crucial for coordinating the M2 macrophage response in the glioblastoma microcompartment. Nonetheless, anti-IL-6 monotherapy only showed modest efficacy in *in vivo* preclinical glioblastoma models (30, 31). Glioblastoma EVs are central to intercellular communication among glioblastoma cells, the local milieu, and peripheral immune cells. Primary cilia are a major organelle in extracellular communication and microenvironment sensing, and we cannot exclude the possibility that primary cilia are involved in GBM EV release, especially considering that vesicles have been shown to be released from primary cilia (15). Whether these primary cilia-derived vesicles are indeed EVs and associated with tumor-mediated immunosuppression, is unknown, and should be an avenue for further investigation. Understanding the contribution of primary cilia to GBM tumor immunosuppression may be pivotal in the development of novel therapies.

### Implications for glioblastoma immunotherapy

Immunotherapy has garnered interest in glioblastoma research in large part due to the revolutionary improvement in survival noted with several systemic cancers. Unfortunately, no immunotherapy treatments have met the efficacy and safety profiles to be adopted for widespread clinical use or FDA approval. It is possible that primary cilia, as the principal organelle in microenvironment sensing and communication, is involved in regulation of both IL-6 and glioblastoma EVs. Primary cilia signaling could be therapeutically targeted, leading to suppression of IL-6, EV packaging and secretion, and other cellular cues such as proliferation and invasion. Thus, the immunosuppressive effects of these soluble factors could be potentially reversed, reconstituting antitumor immunity, rendering glioblastomas more amenable to immunotherapy. Further investigation into the interplay between primary cilia signaling and glioblastoma-mediated immunosuppression will be necessary and may lead to the development of novel ‘ciliotherapeutic’ approaches to glioblastomas.

## Conclusions

Glioblastoma is the most common CNS malignancy. It remains universally fatal with only small gains in survival over the last 3 decades. The tumor is genetically complex with several simultaneously dysregulated pathways. There is also profound local and systemic immunosuppression which limit efficacy of immunotherapy. Understanding the interplay between glioblastoma and its microenvironment is key to developing effective immunotherapies. In this perspectives article, we provide evidence for a role of primary cilia in IL-6 release and immunosuppression. We also suggest a potential role of primary cilia in EV release. There is potential for novel cilia-related therapeutic strategies which would be welcomed addition in the armamentarium against this deadly disease.

## Data availability statement

The raw data supporting the conclusions of the article will be made available by the authors upon request.

## Ethics statement

Ethical approval was not required for the studies on humans in accordance with the local legislation and institutional requirements because only commercially available established cell lines were used.

## Author contributions

ML: Writing – original draft, Writing – review & editing. EW: Data curation, Writing – original draft, Writing – review & editing. FG: Writing – review & editing. LA: Writing – review & editing. M-YJ: Data curation, Writing – review & editing. EB: Supervision, Writing – review & editing. DB: Conceptualization, Supervision, Writing – review & editing.
